# Trajectories of objectively measured physical activity and mood states in older Japanese adults: longitudinal data from the Nakanojo Study

**DOI:** 10.1186/s13030-021-00207-0

**Published:** 2021-02-23

**Authors:** Shuji Inada, Kazuhiro Yoshiuchi, Sungjin Park, Yukitoshi Aoyagi

**Affiliations:** 1 Department of Stress Sciences and Psychosomatic Medicine, Graduate School of Medicine, The University of Tokyo, 7-3-1 Hongo, Bunkyo-ku, Tokyo, Japan; 2 Exercise Sciences Research Group, Tokyo Metropolitan Institute of Gerontology, 35-1 Sakecho, Itabashi-ku, Tokyo, Japan

**Keywords:** Physical activity, Elderly people, Accelerometer, Trajectory analysis, Depression

## Abstract

**Background:**

Japan, like many developed countries, now faces fiscal problems from the escalating health-care expenditures associated with an aging population. Mental health problems such as depression contribute as much to these growing demands as physical disease, and measures to prevent depression are important to controlling costs. There are few longitudinal studies examining the relation between objectively measured physical activity and depressive symptoms. Therefore, the aims of our study were to explore the patterns of change of physical activity in older Japanese adults for 5 years through the use of trajectory analysis and to examine the relation between physical activity trajectories and depressive mood states.

**Main body:**

Ninety-two male and 99 female volunteers aged 65–85 years were asked to equip themselves with an electronic accelerometer with a 60-day storage capacity for at least 5 years. The parameters calculated each July for the 5 years were the average daily step count and the average daily duration of activity > 3 METs (moderate to vigorous physical activity: MVPA). Hospital Anxiety and Depression Scale (HADS) assessed corresponding mood states (HADS-A and HADS-D). Trajectories of the accelerometer data were analyzed and fifth-year HADS-D and HADS-A scores were compared among trajectory groups using an analysis of covariance (ANCOVA) that controlled for baseline scores and for baseline scores and age. Six and five distinct trajectories were identified for daily step count and for daily duration of MVPA, respectively. Using ANCOVA controlling for baseline scores, HADS-D scores differed significantly among trajectory groups classed by daily duration of MVPA (*p* = 0.04), and Tukey’s multiple comparison tests showed significant differences between group 2, whose pattern was stable with the middle duration of MVPA, and group 1, whose pattern was stable with the lowest duration of MVPA (*p* = 0.02), while the results were not significant controlling for both baseline scores and age.

**Conclusions:**

Older people with less MVPA continued to do less MVPA over the 5 years of study, which may be related to a future more depressive mood. Further clinical studies will be necessary to clarify these findings.

## Background

Japan, like many developed countries, now faces fiscal problems from the escalating health-care expenditures associated with an aging population [1]. Mental health problems such as depression contribute as much to these growing demands as physical disease [2], and measures to prevent depression are important to controlling costs.

A systematic review of prospective cohort studies examined the relation between physical activity and risk of depression [3]. Although 49 studies were reviewed in the article, physical activities were assessed by subjective methods, such as a questionnaire on regular exercise, in all studies except one, which may contain a risk of recall bias. In only one study, in which a pedometer was used to assess physical activity objectively, there was no significant relation between physical activity and the incidence of depression [4]. Some cross-sectional studies used accelerometers to assess the relation between physical activity and depression, including our previous study [5–8], but few longitudinal studies have been done to examine the relation between objectively measured physical activity and depressive symptoms.

In addition, physical activity was assessed only at baseline in almost all of the longitudinal studies. Trajectory analysis is a statistical technique that can exploit the existence of latent groups similar in time course of the change of some value. Trajectory analysis is being used for analysis of longitudinal data in the medical context [9, 10]. We can reveal the relation between the pattern of the time change of physical activities and depressive symptoms through the use of trajectory analysis.

Built on our previous cross-sectional study [5], the aims of this study were to explore the patterns of change of physical activity in older Japanese adults for 5 years through the use of trajectory analysis and to examine the relation between physical activity trajectories and depressive mood states.

## Main text

### Methods

#### Participants and procedures

The physical activity trajectories and mood states of 92 male (70.0 ± 3.8 years) and 99 female (69.1 ± 4.4 years) volunteers aged 65–85 years were followed for at least 5 years after they had given their written informed consent to participation in an institutionally approved study. The first of the 5 years was between 2001 and 2003. During this period there was no significant disasters or incidents that would have an impact on the participants’ physical activity. Participants were recruited by flyers at the time of a’ health checkup for the residents of Nakanojo, a medium-sized Japanese town located about 150 km northwest of Tokyo. Its population was around 20,000, and around 30% of the population were aged > = 65 years (around 26% men and 33% women) during the first years. The inclusion criteria were relatively healthy elderly people of > = 65 years of age. The criteria for exclusion were: severe dementia; walking disability; and those who were instructed not to exercise by their physicians. Underlying diseases were as follows: 80 participants were treated for hypertension; 11 for hyperlipidemia; 21 for diabetes; 8 for arrhythmia; and 11 had a history of cancer. An electronic accelerometer with a 60-day storage capacity (Lifecorder; Suzuken Co., Ltd., Nagoya, Japan) attached to a waist-belt on the left side of the body measured the number of steps taken and the intensity of physical activity [1]. The parameters, calculated each July for 5 years, were the average daily step count and the average daily duration of activity > 3 metabolic equivalents (METs) (moderate to vigorous physical activity: MVPA) [1]. Hospital Anxiety and Depression Scale (HADS) were used annually, in July at the conclusion of physical activity measurement, to assess mood states (HADS-A and HADS-D) [11, 12].

#### Statistical analysis

Trajectories of the accelerometer data were analyzed using trajectory analysis by the SAS procedure Proc Traj, which fits a semi-parametric (discrete) mixture model to longitudinal data by the maximum-likelihood method [13]. Baseline ages were compared among trajectory groups using an analysis of variance (ANOVA) followed by the Tukey multiple comparison tests. Proportions of men and women were compared among trajectory groups using the chi-square or Fisher’s exact test. Fifth-year HADS-D and HADS-A scores were compared among trajectory groups using an analysis of covariance (ANCOVA) that controlled for baseline scores and for baseline scores and age followed by the Tukey multiple comparison tests.

## Results

**Fig. 1:**
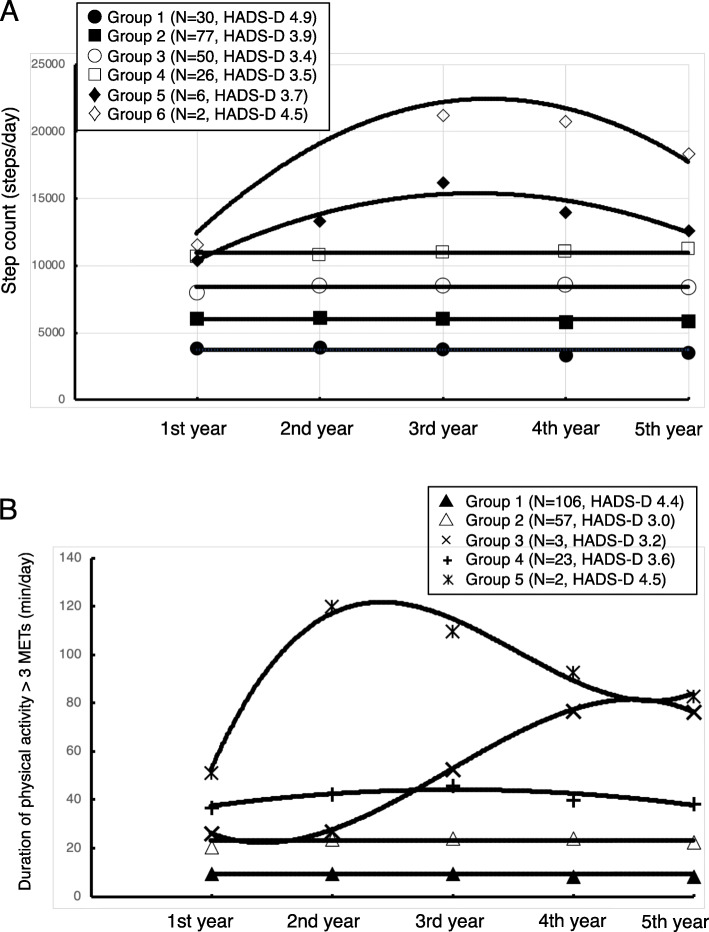
Trajectories of daily step count (**a**) and duration of physical activity > 3 METs (**b**) of elderly Japanese participants over 5 years of observation. The physical activity was measured objectively by the use of an electronic accelerometer. The markers and solid lines indicate the observed data and predicted trajectories, respectively. HADS-D, depression score of Hospital Anxiety and Depression Scalel; METS, metabolic equivalents

Distinct trajectories were identified: 6 for daily step counts (Fig. 1a) and 5 for daily duration of MVPA (Fig. 1b).

With regard to the daily step count, there were significant differences in age when using ANOVA (*p* < 0.001). Tukey multiple comparison tests of daily step count revealed that the age of Group 1 was significantly higher than those of Groups 3 (*p* < 0.01) and 4 (*p* < 0.01) and that the age of the Group 2 was significantly higher than that of Group 3 (*p* < 0.01) (Table 1). With regard to the daily duration of MVPA, there were significant differences in age (*p* < 0.01), and the Tukey multiple comparison test revealed that the age of Group 1 was significantly higher than those of Groups 2 (*p* = 0.02) and 4 (*p* = 0.01) (Table 1). With regard to the proportion of men and women, there was no significant difference among the groups (Table 1).

**Table 1:** Age, proportion of men and women, and HADS scores in the trajectory groups

Tukey’s multiple comparison							
Daily step counts	Group 1 (*n* = 30)	Group 2 (*n* = 77)	Group 3 (*n* = 50)	Group 4 (*n* = 26)	Group 5 (*n* = 6)	Group 6 (*n* = 2)	
Age^*^	71.9 (4.6)	70.4 (4.5)	67.8 (2.7)	67.9 (3.0)	69.7 (4.6)	68.0 (1.4)	Group 1 > Group 3 (*p* < 0.01), Group 1 > Group 4 (*p* < 0.01), Group 2 > Group 3 (p < 0.01)
Number of men	14	34	28	12	3	1	
HADS-D							
Baseline	5.5 (3.2)	4.0 (3.9)	3.8 (2.9)	4.7 (2.9)	4.6 (3.3)	7.5 (2.1)	
Fifth year	4.9 (3.5)	3.9 (3.8)	3.4 (3.0)	3.5 (2.5)	3.7 (1.8)	4.5 (3.5)	
HADS-A							
Baseline	5.8 (2.6)	4.2 (3.1)	4.0 (2.3)	4.8 (3.1)	4.7 (2.8)	4.0 (1.4)	
Fifth year	5.2 (3.4)	3.5 (2.9)	3.0 (2.4)	4.6 (3.2)	2.7 (2.8)	6.5 (2.1)	
Duration of MVPA	Group 1 (*n* = 106)	Group 2 (*n* = 57)	Group 3 (n = 3)	Group 4 (*n* = 23)	Group 5 (*n* = 2)		
Age^†^	70.6 (4.4)	68.6 (3.7)	70.7 (5.5)	67.6 (3.1)	68.0 (1.4)		Group 1 > Group 2 (*p* = 0.02), Group 1 > Group 4 (*p* = 0.01)
Number of men	44	31	2	14	1		
HADS-D							
Baseline	4.5 (3.6)	4.1 (3.4)	2.3 (0.6)	3.9 (2.7)	7.5 (2.1)		
Fifth year^‡^	4.4 (3.8)	3.0 (2.8)	3.2 (1.0)	3.6 (2.8)	4.5 (3.5)		Group 1 > Group 2 (*p* = 0.02)
HADS-A							
Baseline	4.8 (3.0)	4.1 (3.0)	3.3 (1.5)	4.2 (2.5)	4.0 (1.4)		
Fifth year	4.1 (3.2)	3.3 (2.5)	2.7 (2.1)	3.5 (3.1)	6.5 (2.1)		

Age, HADS-D and HADS-A are expressed by mean (SD)

^*^p < 0.01 among the six groups using ANOVA

^†^ *p* < 0.01 among the five groups using ANOVA

^‡^*p* = 0.04 among the five groups using ANCOVA controlling for baseline HADS-D scores, but not significant controlling for age and baseline HADS-D scores

*HADS* Hospital anxiety and depression scale, *HADS-A* Anxiety scores of hospital anxiety and depression scale, *HADS-D* Depression scores of hospital anxiety and depression scale

The HADS-D scores of the fifth year differed significantly among the trajectory groups classified by daily duration of MVPA (*p* = 0.04) using ANCOVA controlling for the baseline scores. Tukey’s multiple comparison tests showed significant differences between groups 1 and 2 (4.4 vs. 3.0, *p* = 0.02) (Table 1). However, there was no significant difference in HADS-A or HADS-D scores of the fifth year using ANCOVA controlling for age and baseline scores.

## Discussion

In the present exploratory study, using trajectory analysis we identified six distinct trajectory groups for daily step count and five groups for daily duration of MVPA. The trajectories for groups other than group 6 for daily step count and other than group 5 for daily duration of MVPA were mostly flat, which means the amount of physical activity did not change in most of the elderly people studied in the natural course of the 5 years.

HADS-D scores differed significantly among the trajectory groups classified by daily duration of activity > 3 METs (*p* = 0.038) using ANCOVA. Tukey’s multiple comparison tests detected a significant difference in HADS-D score between groups 1 and 2 for daily duration of MVPA. These results suggest that people with less MVPA continued to do less MVPA and that they had a more depressive mood at the end of the 5 years of study. In a meta-analysis of previous studies, physical activity had a protective effect against the emergence of depression in the elderly [3]. Therefore, the results of the present study are consistent. In addition, the results of the present study suggest that the daily duration of MVPA might be more important than the daily step count to prevent depressive symptoms.

There were significant differences in age between some groups by daily step count and by duration of MVPA. And the results of ANCOVA controlling for baseline HADS and age were not significant. Therefore, the differences in trajectory patterns might be partly attributable to the differences in age: the daily step count and duration of MVPA in most groups did not decrease during the 5 years. The effect of aging should be clarified in future studies with larger sample sizes.

There are some limitations in the present study. First, the sample size was small, therefore we did not have sufficient power to make definite conclusions on differences between groups. Second, we did not measure some confounding factors, such as lifestyle or personality. Therefore, future studies with more participants will be necessary to prove how the trajectory of physical activity affects depressive symptoms and also to investigate the relation between the trajectories of physical activity and the trajectories of mood states. In addition, the present study was exploratory and done only with generally healthy participants. Therefore, the findings should be interpreted with caution and cannot be expanded to clinical significance.

## Conclusions

Older people with less MVPA continued to do less MVPA over the 5 years of study, which might be related to a future more depressive mood. Further clinical studies will be necessary to clarify these findings.

## Data Availability

We are not able to share our data because sharing data is not permitted by our hospital or the ethics committee.

## Abbreviations

ANOVA: Analysis of variance
ANCOVA: Analysis of covariance
HADS-A: Hospital Anxiety and Depression Scale anxiety score
HADS-D: Hospital Anxiety and Depression Scale depression score
METs: Metabolic equivalents
MVPA: Moderate to vigorous physical activity
